# Using rhetorical appeals to credibility, logic, and emotions to increase your persuasiveness

**DOI:** 10.1007/s40037-018-0420-2

**Published:** 2018-05-07

**Authors:** Lara Varpio

**Affiliations:** Department of Medicine, Uniformed Services University of the Health Sciences, Bethesda, MD USA

In the Writer’s Craft section we offer simple tips to improve your writing in one of three areas: Energy, Clarity and Persuasiveness. Each entry focuses on a key writing feature or strategy, illustrates how it commonly goes wrong, teaches the grammatical underpinnings necessary to understand it and offers suggestions to wield it effectively. We encourage readers to share comments on or suggestions for this section on Twitter, using the hashtag: #how’syourwriting?

Scientific research is, for many, the epitome of objectivity and rationality. But, as Burke reminds us, conveying the meaning of our research to others involves persuasion. In other words, when I write a research manuscript, I must construct an argument to persuade the reader to accept *my rationality*.

While asserting that scientific findings must be persuasively conveyed may seem contradictory, it is simply a consequence of how we conduct research. Scientific research is a social activity centred on answering challenging questions. When these questions are answered, the solutions we propose are just that—propositions. Our solutions are accepted by the community until another, better proposition offers a more compelling explanation. In other words, everything we know is accepted *for now* but not *forever*.

This means that when we write up our research findings, we need to be persuasive. We must convince readers to accept our findings and the conclusions we draw from them. That acceptance may require dethroning widely held perspectives. It may require having the reader adopt new ways of thinking about a phenomenon. It may require convincing the audience that other, highly respected researchers are wrong. Regardless of the argument I want the reader to accept, I have to persuade the reader to agree with *me*.

Therefore, being a successful researcher requires developing the skills of persuasion—the skills of a rhetorician. Fortunately for the readers of *Perspectives on Medical Education, The Writer’s Craft* series offers a treasure trove of rhetorical tools that health professions education researchers can mine.

A primary lesson of rhetoric was developed by Aristotle. He studied rhetoric analytically, investigating all the means of persuasion available in a given situation. He identified three appeals at play in all acts of persuasion: ethos, logos and pathos. The first is focused on the author, the second on the argument, the third on the reader. Together, they support effective persuasion, and so can be harnessed by researchers to powerfully convey the meaning of their research.

## Ethos

Ethos is the appeal focused on the writer. It refers to the character of the writer, including her credibility and trustworthiness. The reader must be convinced that the author is an authority and merits attention. In scientific research, the author must establish her credibility as a rigorous and expert researcher. Much of an author’s ethos, then, lies in using well-reasoned and justified research methodologies and methods. But, a writer’s credibility can be bolstered using a number of rhetorical techniques including *similitude *and *deference*.

*Similitude* appeals to similarities between the author and the reader to create a sense of mutual identification. Using pronouns like *we* and *us, *the writer reinforces commonality with the reader and so encourages a sense of cohesion and community. To illustrate, consider the following:

While burnout continues to plague *our residents*, medical educators have yet to identify the root causes of this problem. *We* owe it to *our residents* to delve into this area of inquiry to secure their wellbeing over their lifetime of clinical service.

versus:

While burnout continues to plague *residents*, medical educators have yet to identify the root causes of this problem. *Medical educators* owe it to *their residents* to delve into this area of inquiry to secure their wellbeing over their lifetime of clinical service.

In the first sentence, the author aligns herself with the community of medical educators involved in residency education. The writer is part of the *we *who has to support residents. She makes the burnout problem something *she and the reader *are both called upon to address. In the second sentence, the author separates herself from this community of educators. She creates social distance between herself and the reader, and thus places the burden of resolving the problem more squarely on the shoulders of the reader than herself.

Both phrasings are equally correct, grammatically. One creates social connection, the other social distance.

*Deference *is a way for the author to signal respect for others, and personal humility. The writer can demonstrate deference by using phrases such as *in my opinion*, or through the use of adjectives (e.g., Smith *rigorously* studied) or adverbs (e.g., the *important *work by Jones). For example:

The *thoughtful* research conducted by Jane Doe et al. suggests that resident burnout is more prevalent among those learners who were shamed by attending physicians. Echoing the calls of others [1], we contend that *this work should be extended* to also consider the role of fellow learners as potential contributors to resident experiences of burnout.

In this sentence, the author does not present Jane Doe and colleagues as weak researchers, nor as developing findings that should be rejected. Instead, it shows deference to these researchers by acknowledging the quality of their research and a willingness to build on the foundation provided by their findings. (Note how the author also builds ethos via similitude with other scholars by calling the reader’s attention to the fact that other researchers have also called for more research on the author’s suggested extension of Doe’s work).

Readers pick up on the respect authors pay to other researchers. Being rude or unkind in our writing rarely achieves anything except reflecting poorly on the writer.

In sum, as my grandmother used to say: ‘You’ll slide farther on honey than gravel.’ Establishing similitude and showing deference helps to establish your ethos as an author. They help the writer make honey, not gravel.

## Logos

Logos is the rhetorical appeal that focuses on the argument being presented by the author. It is an appeal to rationality, referring to the clarity and logical integrity of the argument. Logos is, therefore, primarily rooted in the reasoning that holds different elements of the manuscript’s argument together. Do the findings logically connect to support the conclusion being drawn? Are there errors in the author’s reasoning (i.e., logical fallacies) that undermine the logic presented in the manuscript? Logical fallacies will undercut the persuasive power of a manuscript. Authors are well advised to spend time mapping out the premises of their arguments and how they logically lead to the conclusions being drawn, avoiding common errors in reasoning (see Purdue’s on-line writing lab [2] for 12 of the most common logical fallacies that plague authors, complete with definitions and examples).

However, logos is not merely contained in the logic of the argument itself. Logos is only achieved if the reader is able to follow the author’s logic. To support the reader’s ability to process the logical argument presented in the manuscript, authors can use *signposting. *Signposting is often accomplished via words (e.g., *first, next, specifically, alternatively, also, consequently, *etc.) and phrases (e.g., *as a result, and yet, for example, in conclusion, *etc.) that help the reader to follow the line of reasoning as it moves through the manuscript. Signposts indicate to the reader the structure of the argument to come, where they are in the argument at the moment, and/or what they can expect to come next. Consider the following sentence from one of my own manuscripts. This is the last sentence in the Introduction [3]:

*This study addresses these gaps by investigating the following questions:*

1. How often are residents taught informally by physicians and nurses in clinical settings?
2. What competencies are informally taught to residents by physicians and nurses?
3. What teaching techniques are used by physicians and nurses to deliver informal education?

At the end of the Introduction, this sentence offers a map to the reader of how the paper’s argument will develop. The reader can now expect that the manuscript will address each of these questions, in this order. I could also use large-scale signposting, such as sub-headings in the Results, to organize the reading of data related to each of these questions. In the Discussion, I can use small-scale signpost terms and phrases (i.e., *however, in contrast, in addition, finally, *etc.) to help the reader follow the progression of the argument I am presenting*.*

I must offer one word of caution here: be sure to use your signposts precisely. If not, your writing will not be logically developed and you will weaken the logos at work in the manuscript. For instance, *however *signposts a contrasting or contradicting idea:

I enjoy working with residents; *however*, I loathe completing in-training evaluation reports.

If the writer uses the wrong signpost, the meaning of the sentence falls apart, and so does the logos:

I enjoy working with residents; *alternatively*, I loathe completing in-training evaluation reports.

*Alternatively *indicates a different option or possibility. This sentence does not present two different alternatives; it presents two contrasting ideas. Using *alternatively *confuses the meaning of the sentence, and thus impairs logos.

With clear and precise signposting, the reader will easily follow your argument across the manuscript. This supports the logos you develop as you guide the reader to your conclusions.

## Pathos

Pathos is the rhetorical appeal that focuses on the reader. Pathos refers to the emotions that are stirred in the reader while reading the manuscript. The author should seek to trigger specific emotional reactions in their writing. And, yes, there is room for emotions in scientific research articles. Some of my favourite manuscripts in *The Writer’s Craft *series are those that help authors elicit specific emotions from the reader.

For instance, in *Joining the conversation: the problem/gap/hook heuristic *Lingard highlights the importance of ‘hooking’ your audience. The hook ‘convinces readers that this gap [in the current literature] is of consequence’ [4]. The author must persuade the reader that the argument is important and worthy of the reader’s attention. This is an appeal to the readers’ emotions.

Another example is found in *Bonfire red titles. *As Lingard explains, the title of your manuscript is ‘advertising for what is inside your research paper’ [5]. The title must attract the readers’ attention and create a desire within them to read your manuscript. Here, again, is pathos in action in a scientific research paper: grab the reader’s attention from the very first word of the title.

Beyond those already addressed in *The Writer’s Craft *series, another rhetorical technique that appeals to the emotions of the reader is the strategic use of *God-terms *[1]*. *Burke defined God-terms as words or phrases that are ‘the ultimates of motivation,’ embodying characteristics that are fundamentally valued by humans. To use an analogy from card games (e.g., bridge or euchre), God-terms are like emotional trump cards. God-terms like *freedom, justice, *and *duty *call on shared human values, trumping contradictory feelings. By alluding to God-terms in our research, we increase the emotional appeal of our writing. Let us reconsider the example from above:

While burnout continues to plague our residents, medical educators have yet to identify the root causes of this problem. *We owe it* to our residents to delve into this area of inquiry to secure their wellbeing over their *lifetime of clinical service*.

Here, the author reminds the reader that residents will be *in service* as physicians for their lifetime, and that we have a *duty* (i.e., *we owe it*) to support them in that calling to meet the public’s healthcare needs. Invoking the God-terms of *service *and *duty, *the writer taps into the reader’s sense of responsibility to support these learners.

It is important not to overplay pathos in a scientific research paper—i.e., readers are keenly intelligent scholars who will easily identify emotional exaggeration. Consider this variation on the previous example:

While burnout continues to *ruin the lives* of our residents, medical educators have neglected to identify the root causes of this problem. *We have a moral obligation* to our residents to delve into this area of inquiry to secure their wellbeing over their lifetime of clinical service.

This rephrasing is likely to create a sense of unease in the reader because of the emotional exaggerations it uses. By over-amplifying the appeals to emotion, this rephrasing elicits feelings of refusal and rejection in the reader. Instead of drawing the reader in, it pushes the reader away. When it comes to pathos, a light hand is best.

## In summary

Peter Gould famously stated: ‘data can never speak for themselves’ [6]. Researchers must explain them. In that explaining, we endeavour to convince the audience that our propositions should be accepted. While the science in our research is at the core of that persuasion, there are techniques from rhetoric that can help us convince readers to accept our arguments. Ethos, logos and pathos are appeals that, when used intentionally and judiciously, can buoy the persuasive power of your manuscripts.

## Disclaimer

The views expressed herein are those of the authors and do not necessarily reflect those of the United States of America’s Department of Defense or other federal agencies.
